# Fighting respiratory diseases: divided efforts lead to weakness

**DOI:** 10.1590/S1806-37132014000300001

**Published:** 2014

**Authors:** Rogelio Pérez-Padilla, Rafael Stelmach, Manuel Soto-Quiroz, Álvaro Augusto Cruz

**Affiliations:** National Institute of Respiratory Diseases, Mexico City, Mexico; Pulmonary Division, Heart Institute, University of São Paulo Hospital das Clínicas, São Paulo, Brazil; Hospital Nacional de Niños, San José, Costa Rica; Center of Excellence in Asthma, Federal University of Bahia, Salvador, Brazil

Various respiratory diseases have been leading causes of death and morbidity over time, and that could be expected considering the huge interface between the respiratory system and the often hostile environment. The respiratory system filters almost 100,000 liters of air in an adult every day. The relevance of the burden of respiratory diseases has been recently emphasized by publications of the major respiratory societies in the world.^(^ ^1^ ^-^ ^4^ ^)^

Tuberculosis, the white plague and the origin of pulmonology, was an epidemic and still causes a great number of deaths and morbidity, due to public health neglect and the AIDS epidemic. In addition, inefficacious treatment is an increasing problem, with the presence of mycobacteria that are resistant to all existing drugs. Acute respiratory infections (ARIs), especially pneumonia, cause deaths in all ages, being especially relevant in children in developing countries. ARIs are the most common cause of outpatient consultation in the majority of countries. Tuberculosis and ARIs will most likely remain as leading health problems in the near future.^(^ ^5^ ^,^ ^6^ ^)^ More recently, we have had to face chronic noncommunicable diseases in the rise, which was highlighted by the World Health Organization (WHO) in 2005.^(^ ^7^ ^)^ Bronchial asthma affects approximately 10% of the world population, with great variations among the countries according to the International Study of Asthma and Allergy in Childhood,^(^ ^8^ ^-^ ^16^ ^)^ causing morbidity, impairment, poor quality of life, and substantial health expenditures, being on the rise in various countries. Although deaths are uncommon in asthma, virtually all ARIs can be considered preventable, and they decrease progressively with the proper treatment of patients. In addition, COPD affects from 8-20% of the adult populations in five cities of Latin America^(^ ^17^ ^)^ and is the third leading cause of death in the world. The disease, which ranks third or fourth among the top illnesses in various countries, has high morbidity and generates remarkably high health expenditures. Lung cancer is also on the rise, being among the ten leading causes of death in 2010. Recently, a successful detection program in high-risk subjects based on chest CT scanning has been described.^(^ ^18^ ^)^

These "big five" respiratory diseases (ARI, tuberculosis, asthma, COPD, and lung cancer), due to their extremely high combined burden, should receive more attention by health and economy authorities in developing countries, in accordance with the recommendations of a recent report by the Forum of the International Respiratory Societies.^(^ ^4^ ^)^ In fact, if we group the codes of all respiratory diseases in the International Classification of Diseases, 10th revision (ICD-10) together, their burden and mortality are quite similar to those of cardiovascular diseases and cancer, and much higher than those of diabetes, which usually receives abundant funds for health care, research, and health promotion activities. Except for some priority regarding tuberculosis, respiratory diseases are often neglected in developing countries. In 2005 and 2008, respectively, 14.7% and 13.4% of all deaths had acute or chronic respiratory origin in Mexico,^(^ ^19^ ^)^ which was close to the proportion of all cancer and cardiovascular-related deaths. This happens frequently in other countries.^(^ ^19^ ^)^

The results of various studies have markedly increased the knowledge of the natural history of asthma and showed the relationship between persistent asthma during childhood and the development of chronic lung disease. It is relevant that prematurity is associated with significant decreases in lung function early in life, which influences the development of chronic lung disease later.

It is relevant to analyze why this is happening. One factor that contributes to the problem is the underestimation of the burden of respiratory diseases, and this is related to the imperfections of the ICD-10, which is based on different perspectives: by organs or systems (respiratory diseases for example, the J codes), by mechanisms of disease (infections, in which ARIs and tuberculosis are inserted), but also by the period of life of the patients (neonatal diseases, infectious or respiratory, are in a separate section, as well as obstetrical problems). Various diseases overlap organs or systems and have to be classified in one of the major codes. For example, pulmonary thromboembolism, which is one of the major causes of mortality in pulmonary medicine, is classified in the cardiovascular disease group. Cancer is a very heterogeneous group, and lung cancer is classified in the C34 codes, although it is more closely related to COPD, due to smoking-a shared risk factor, than to other neoplasias. 

The formation of the ICD-10 groups also depends on the emphasis and priorities identified by the WHO and other health care organizations, which explains the existence of a group for neonatal and obstetrical diseases, regardless of the organ affected or the mechanism of disease.

Obstructive sleep apnea syndrome (OSAS) is also extremely common, since it affects 2-4% of the population.^(^ ^20^ ^,^ ^21^ ^)^ In childhood, it appears because of tonsil and adenoid hypertrophy; in adulthood and in the elderly, obesity is the leading risk factor.^(^ ^22^ ^)^ OSAS requires permanent treatment with continuous positive airway pressure, increases the risk of accidents and learning difficulties, reduces quality of life, and leads to metabolic and cardiovascular complications.^(^ ^23^ ^)^ Hypoxemia is relevant in cities at moderate or high altitude, which are common in Latin America. In Mexico City (2,240 m above sea level), 6% of individuals aged 40 years or older present with SaO2 ≤ 88%^(^ ^24^ ^)^; however, less than 8% of these individuals have been prescribed oxygen therapy. 

Official Ministries and Departments of Health in various countries, as well as the WHO, tend to follow the ICD-10 compartmentalization. For example, there are different departments or divisions for tuberculosis, ARIs, and chronic respiratory diseases. This segregation conflicts with the everyday reality in primary health care,^(^ ^25^ ^)^ which is relevant and shall be integrated and multifunctional.^(^ ^26^ ^)^ Integrality and multifunctionality are difficult to achieve with separate programs for common respiratory diseases. In addition, patients with acute or chronic lung diseases seek primary care because of a limited variety of respiratory symptoms. Guidelines for the treatment of respiratory diseases are varied, and before using them, health professionals have to decide what is appropriate for the patient, knowing that overlapping of acute and chronic diseases is common, such as asthma or COPD and acute respiratory infection, or acute respiratory infection and lung cancer. 

The training of health care personnel, both in primary care and in specialties, disregards the ICD-10 classification and the WHO recommendations and includes acute, chronic, communicable, and noncommunicable diseases all together, as it happens in the real world. 

Cumulative exposure to tobacco (by active or second-hand smoking), occupational fumes, and indoor and outdoor air pollution are known risks for various respiratory diseases. With aging, these risks are increased by higher prevalences of obesity and diabetes, lack of vaccination for preventable infections, and other traditional causes of death, such as tuberculosis.

National respiratory programs are uncommon when compared with programs for other diseases, despite their relevance. When they exist, they are separated in accordance with the ICD codes or the WHO departments; however, we need them to be integrated in primary health care. A patient with chronic cough and phlegm requires an investigation for tuberculosis; nevertheless, most of them do not have tuberculosis and are finally sent somewhere else. More importantly, that patient has a health problem, which requires evaluation and treatment, regardless of whether it is an acute, subacute, or chronic condition. This should be the role of an integrated program, including medical attention for infections, cancer, asthma, and COPD, regardless of their ICD-10 codes or the organization of departments in Ministries of Health or the WHO. The compartmentalization of public heath care initiatives against respiratory conditions in Latin America and in other countries might result in the weakness of the whole system. Just as an example, COPD causes more deaths than AIDS, breast cancer, cervical cancer, and prostate cancer together in Mexico and in Latin America. There are national cancer programs in many countries, but no COPD programs that cover all levels of health care. 

The Practical Approach to Lung Health (PAL), a WHO program,^(^ ^27^ ^-^ ^31^ ^)^ proposes integration in primary health care by using as its first step the syndromic diagnosis, starting in the tuberculosis clinics that are present everywhere, and taking care of all of the individuals screened for tuberculosis with negative tests. This is a sound program, since it expands with funding and training from existing assets and reinforces the tuberculosis program, which is abandoned or underfunded in various places. An integrated program not only deals with neglected diseases, such as COPD, asthma, and lung cancer, but also reinforces existing programs, such as tuberculosis programs and, in some countries, acute respiratory disease programs. Programs similar to PAL have been able to reduce the use of antibiotics and symptomatic medications, as well as to resolve increasingly health problems in primary health care, reinforcing the fight against tuberculosis. Single disease programs have shown that asthma and COPD patients receive better health care, impairment decreases, and fewer hospitalizations occur, provided that access to drugs is secured.^(^ ^32^ ^-^ ^34^ ^)^ In addition, this type of programs reduces deaths and health expenditures in asthma and COPD,^(^ ^35^ ^)^ which would be included in integrated programs.

Prevention is definitely the key, and anti-tobacco regulations considerably help. The health care costs of tobacco-related diseases are considerably higher than are tobacco product taxation. Campaigns against other respiratory risk factors-outdoor and indoor air pollution, occupational risks, obesity, among others-are required but uncommon. Anti-tobacco advice and medications, which are part of the effective recommendations by the WHO, should be included in an integrated respiratory program. 

One-disease national programs have various potential problems emphasized by the WHO: difficulties in long-term sustainability and in the transition to multifunctional integrated programs; duplicity of supervision and training; and possible discrimination against patients outside the program. Integrated programs similar to PAL require adaptations to the necessities of the area or the country in which they are implemented. For example, in South Africa, an integrated program includes the diagnosis and treatment of HIV and AIDS, which are leading local health problems. 

In summary, we would do a great service to patients with respiratory diseases if we offer them integrated primary health care programs similar to what PAL proposes. We would have better grounds to compete for funding if the proposal is part of a successful strategy that includes integration. The way things are at the moment-the fragmented, underfunded organization of health care, regardless of its origins-might be an obstacle rather than an asset in order to improve respiratory health.
