# Supplementary material for: Fighting respiratory diseases: divided efforts lead to weakness
Source: J Bras Pneumol. 2014 May-Jun;40(3):207–10. doi: 10.1590/S1806-37132014000300001 (PMC4109190; doi:10.1590/S1806-37132014000300001)
Supplement: Translation

# Translation: Combate a doenças respiratórias: esforços divididos levam ao enfraquecimento

**Authors:** Rogelio Pérez-Padilla, Rafael Stelmach, Manuel Soto-Quiroz, Álvaro Augusto Cruz

**Affiliations:** Instituto Nacional de Doenças Respiratórias, Cidade do México, México; Divisão de Pneumologia, Hospital das Clínicas, Universidade de São Paulo, São Paulo (SP) Brasil; Hospital Nacional de Crianças, São José, Costa Rica; Núcleo de Excelência em Asma, Universidade Federal da Bahia, Salvador (BA) Brasil

Diversas doenças respiratórias têm sido importantes causas de morte e morbidade ao longo do tempo, o que se poderia esperar, considerando-se a enorme interface entre o sistema respiratório e o ambiente muitas vezes hostil. Em adultos, o sistema respiratório filtra quase 100.000 litros de ar todos os dias. A relevância da carga das doenças respiratórias foi recentemente enfatizada por publicações das principais sociedades respiratórias do mundo.^(^ ^1^ ^-^ ^4^ ^)^

A tuberculose, peste branca e origem da pneumologia, foi uma epidemia e ainda causa grande número de mortes e morbidade, em razão da negligência da saúde pública e da epidemia de AIDS. Além disso, o tratamento ineficaz é um problema crescente, com a presença de micobactérias que são resistentes a todos os medicamentos existentes. As infecções respiratórias agudas (IRAs), especialmente a pneumonia, causam mortes em todas as idades, sendo especialmente relevantes em crianças de países em desenvolvimento. As IRAs são a causa mais frequente de consultas ambulatoriais na maioria dos países. A tuberculose e as IRAs provavelmente continuarão sendo os principais problemas de saúde em um futuro próximo.^(^ ^5^ ^,^ ^6^ ^)^ Mais recentemente, tivemos que enfrentar doenças crônicas não transmissíveis em ascensão, o que foi destacado pela Organização Mundial da Saúde (OMS) em 2005.^(^ ^7^ ^)^ A asma brônquica afeta aproximadamente 10% da população mundial, com grandes variações entre os países de acordo com o *International Study of Asthma and Allergy in Childhood*,^(^ ^8^ ^-^ ^16^ ^)^ causando morbidade, comprometimento, baixa qualidade de vida e gastos substanciais em saúde, estando em ascensão em diversos países. Embora mortes sejam incomuns em asma, praticamente todas as IRAs podem ser consideradas evitáveis, e elas diminuem progressivamente com o tratamento adequado dos pacientes. Além disso, a DPOC afeta de 8-20% da população adulta de cinco cidades da América Latina^(^ ^17^ ^)^ e é a terceira principal causa de morte no mundo. A doença, que ocupa a terceira ou quarta posição entre as principais enfermidades em diversos países, apresenta alta morbidade e gera gastos sobremaneira elevados em saúde. O câncer de pulmão também está em ascensão, encontrando-se entre as dez principais causas de morte em 2010. Recentemente, foi descrito um programa bem-sucedido de detecção em indivíduos de alto risco com base na TC de tórax.^(^ ^18^ ^)^

Essas "cinco grandes" doenças respiratórias (IRA, tuberculose, asma, DPOC e câncer de pulmão), em razão de sua elevadíssima carga combinada, devem receber mais atenção por parte das autoridades econômicas e de saúde de países em desenvolvimento, de acordo com as recomendações de um relatório recente do *Forum of International Respiratory Societies.* ^(^ ^4^ ^)^ Na verdade, aos agruparmos os códigos de todas as doenças respiratórias na Classificação Internacional de Doenças, 10ª revisão (CID-10), observamos que sua carga e mortalidade são bastante semelhantes às das doenças cardiovasculares e do câncer, e muito maior do que as do diabetes, que geralmente recebe recursos abundantes para cuidados de saúde, pesquisa e atividades de promoção à saúde. Exceto por certa prioridade quanto à tuberculose, as doenças respiratórias são muitas vezes negligenciadas em países em desenvolvimento. Em 2005 e 2008, respectivamente, 14,7% e 13,4% do total de mortes tiveram origem respiratória aguda ou crônica no México,^(^ ^19^ ^)^ o que ficou próximo da proporção do total de mortes relacionadas a câncer e doenças cardiovasculares. Isso acontece frequentemente em outros países.^(^ ^19^ ^)^

Os resultados de diversos estudos têm aumentado acentuadamente o conhecimento sobre a história natural da asma e mostrado o relacionamento entre a asma persistente na infância e o desenvolvimento de doença pulmonar crônica. É relevante que a prematuridade está associada à diminuição significativa da função pulmonar no início da vida, o que influencia o desenvolvimento de doença pulmonar crônica mais tarde.

É relevante analisar porque isso está acontecendo. Um fator que contribui para o problema é a subestimação da carga das doenças respiratórias, e isso está relacionado às imperfeições da CID-10, a qual se baseia em diferentes perspectivas: por órgãos ou sistemas (doenças respiratórias, por exemplo, códigos J), por mecanismos da doença (infecções, onde se inserem as IRAs e a tuberculose), mas também pelo período de vida dos pacientes (doenças neonatais, infecciosas ou respiratórias, estão em uma seção separada, bem como os problemas obstétricos). Diversas doenças se sobrepõem em órgãos e sistemas e devem ser classificadas em um dos códigos principais. Por exemplo, o tromboembolismo pulmonar, que é uma das principais causas de mortalidade em pneumologia, é classificado no grupo de doenças cardiovasculares. O câncer constitui um grupo muito heterogênio, e o câncer de pulmão é classificado nos códigos C34, embora esteja mais intimamente relacionado à DPOC, em razão do tabagismo - um fator de risco em comum, do que a outras neoplasias.

A formação dos grupos da CID-10 também depende da ênfase e das prioridades identificadas pela OMS e outras organizações de saúde, o que explica a existência de um grupo para doenças neonatais e obstétricas, independentemente do órgão afetado ou do mecanismo da doença.

A síndrome da apneia obstrutiva do sono (SAOS) também é extremamente comum, uma vez que afeta 2-4% da população.^(^ ^20^ ^,^ ^21^ ^)^ Na infância, surge por hipertrofia das amígdalas e das adenoides; na idade adulta e na velhice, a obesidade é o principal fator de risco.^(^ ^22^ ^)^ A SAOS requer tratamento permanente com pressão positiva contínua nas vias aéreas, aumenta o risco de acidentes e de dificuldades de aprendizagem, reduz a qualidade de vida e leva a complicações metabólicas e cardiovasculares.^(^ ^23^ ^)^ A hipoxemia é relevante em cidades em altitudes moderadas ou grandes, as quais são comuns na América Latina. Na cidade do México (2.240 m acima do nível do mar), 6% dos indivíduos com idade igual ou superior a 40 anos apresentam SaO_2_ ≤ 88%(24); porém, menos de 8% desses indivíduos receberam prescrição de oxigenoterapia.

Ministérios e Departamentos de Saúde Oficiais de diversos países, bem com a OMS, tendem a seguir a compartimentação da CID-10. Por exemplo, existem departamentos ou divisões diferentes para a tuberculose, as IRAs e as doenças respiratórias crônicas. Essa segregação entra em conflito com a realidade cotidiana na atenção primária à saúde,^(^ ^25^ ^)^ a qual é relevante e deve ser integrada e multifuncional.^(^ ^26^ ^)^ A integralidade e a multifuncionalidade são difíceis de serem atingidas com programas diferentes para doenças respiratórias comuns. Além disso, pacientes com doenças agudas e crônicas buscam a atenção primária devido a uma variedade limitada de sintomas respiratórios. As diretrizes para o tratamento das doenças respiratórias são variadas, e antes de utilizá-las, os profissionais de saúde devem decidir o que é apropriado para o paciente, sabendo que a sobreposição de doenças agudas e crônicas é comum, como a asma ou DPOC e infecção respiratória aguda ou infecção respiratória aguda e câncer de pulmão.

O treinamento do pessoal da área de saúde, tanto na atenção primária quanto nas especialidades, desconsidera a CID-10 e as recomendações da OMS e inclui as doenças agudas, crônicas, transmissíveis e não transmissíveis todas juntas, como acontece no mundo real.

A exposição cumulativa ao tabaco (por tabagismo ativo ou passivo), fumos ocupacionais e poluição do ar interior e exterior são fatores de risco conhecidos para diversas doenças respiratórias. Com o envelhecimento, esses riscos são aumentados por maiores prevalências de obesidade e diabetes, falta de vacinação para infecções evitáveis e outras causas tradicionais de morte, como a tuberculose.

Programas nacionais de doenças respiratórias são incomuns quando comparados a programas para outras doenças, apesar de sua relevância. Quando existem, são separados de acordo com os códigos da CID ou os departamentos da OMS; porém, precisamos que eles sejam integrados na atenção primária à saúde. Um paciente com tosse crônica e catarro requer investigação para tuberculose; contudo, a maioria deles não tem tuberculose e é finalmente enviada para outro lugar. Mais importante ainda, esse paciente tem um problema de saúde, o qual requer avaliação e tratamento, independentemente de se tratar de uma condição aguda, subaguda ou crônica. Esse deve ser o papel de um programa integrado, incluindo atendimento médico para infecções, câncer, asma e DPOC, independentemente dos seus códigos da CID-10 ou da organização de departamentos de Ministérios da Saúde ou da OMS. A compartimentação das iniciativas de saúde pública contra condições respiratórias na América Latina e em outros países pode resultar no enfraquecimento de todo o sistema. Apenas como exemplo, a DPOC causa mais mortes do que a AIDS, o câncer de mama, o câncer cervical e o câncer de próstata juntos no México e na América Latina. Existem programas nacionais de câncer em muitos países, mas não programas de DPOC que cubram todos os níveis de atenção à saúde. 

A estratégia *Practical Approach to Lung Health* (PAL, Abordagem Prática à Saúde Pulmonar), da OMS,^(^ ^27^ ^-^ ^31^ ^)^ propõe integração na atenção primária á saúde utilizando, como primeiro passo, o diagnóstico sindrômico, começando nas clínicas de tuberculose presentes em toda parte e cuidando de todos os indivíduos rastreados para tuberculose com testes negativos. Esse é um bom programa, uma vez que se expande com financiamento e treinamento a partir de capital existente e reforça o programa de tuberculose, o qual é abandonado ou subfinanciado em diversos locais. Um programa integrado não apenas lida com doenças negligenciadas, como DPOC, asma e câncer de pulmão, mas também reforça programas existentes, como programas de tuberculose e, em alguns países, programas de doenças respiratórias agudas. Programas semelhantes à estratégia PAL têm sido capazes de reduzir o uso de antibióticos e medicações sintomáticas, bem como de resolver cada vez mais problemas de saúde na atenção primária à saúde, reforçando a luta contra a tuberculose. Programas centrados em uma única doença têm mostrado que pacientes asmáticos e pacientes com DPOC recebem melhores cuidados de saúde, o comprometimento diminui e ocorrem menos internações, desde que o acesso aos medicamentos seja assegurado.^(^ ^32^ ^-^ ^34^ ^)^ Além disso, esse tipo de programa reduz as mortes e os gastos em saúde em asma e DPOC,^(^ ^35^ ^)^ as quais seriam incluídas em programas integrados.

A prevenção é definitivamente a chave, e medidas antitabaco ajudam consideravelmente. Os custos com cuidados de saúde de doenças relacionadas ao tabaco são consideravelmente maiores do que a tributação dos produtos do tabaco. Campanhas contra outros fatores de risco respiratório - poluição do ar interior e exterior, riscos ocupacionais, obesidade, entre outros - são necessárias, mas incomuns. Aconselhamento e medicamentos antitabaco, os quais são parte das recomendações efetivas da OMS, devem ser incluídos em um programa integrado de doenças respiratórias.

Programas nacionais centrados em uma doença têm diversos problemas potenciais enfatizados pela OMS: dificuldades na sustentabilidade em longo prazo e na transição para programas integrados multifuncionais; duplicidade de supervisão e treinamento; e possível discriminação contra pacientes fora do programa. Programas integrados semelhantes à estratégia PAL requerem adaptações às necessidades da área ou país em que estão sendo implantados. Por exemplo, na África do Sul, um programa integrado inclui o diagnóstico e o tratamento de HIV e AIDS, que são importantes problemas de saúde locais.

Em suma, prestaríamos um grande serviço aos pacientes com doenças respiratórias se lhes oferecermos programas integrados de atenção primária à saúde semelhantes ao que a estratégia PAL propõe. Teríamos melhores fundamentos para competir por financiamento se a proposta for parte de uma estratégia bem-sucedida que inclua a integração. O atual estado das coisas - a organização fragmentada e subfinanciada da atenção à saúde, independentemente de suas origens - pode ser um obstáculo em vez de um capital para a melhoria da saúde respiratória.
